# DUSP9 is Up-Regulated and Promotes Tumor Progression in Head and Neck Squamous Cell Carcinoma

**DOI:** 10.7150/jca.110597

**Published:** 2025-07-24

**Authors:** Yuzhe Hu, Yue Li, Chenfeng Jiang, Wenling Han, Xiaohong Chen, Pingzhang Wang, Hongbo Xu

**Affiliations:** 1 Department of Immunology, NHC Key Laboratory of Medical Immunology (Peking University), Medicine Innovation Center for Fundamental Research on Major Immunology-related Diseases, School of Basic Medical Sciences, Peking University Health Science Center, Beijing, 100191, China.; 2 Peking University Center for Human Disease Genomics, Beijing, 100191, China.; 3 Department of Otolaryngology-Head and Neck Surgery, Key Laboratory of Otolaryngology Head and Neck Surgery, Beijing Tongren Hospital, Capital Medical University, Beijing, 100730, China.; 4 People's Hospital of Ningxia Hui Autonomous Region, Ningxia Medical University, Ningxia, 750002, China.

**Keywords:** DUSP9, HNSCC, proliferation, migration, tumor infiltration

## Abstract

Head and neck squamous cell carcinoma (HNSCC) ranks among the most prevalent malignancies with a poor prognosis. The underlying mechanisms driving HNSCC carcinogenesis are not fully elucidated. In this study, we identified dual specificity phosphatase 9 (DUSP9) as a carcinogenic factor in HNSCC development. According to the public data, DUSP9 was significantly up-regulated in HNSCC tumor tissues compared to normal tissues, confirmed by clinical data and single-cell RNA sequencing (scRNA-seq) data. Survival analysis revealed that high levels of DUSP9 expression contribute to poor prognosis in HNSCC patients. Knockdown of DUSP9 decreased, but overexpression of DUSP9 increased the proliferation and migration of HNSCC cells. ScRNA-seq data analysis suggested that DUSP9 was selectively expressed in tumor cells, with negligible expression in immune cells and stromal cells, and showed an elevated trend from primary tissues to metastatic tissues. Enrichment analyses of DUSP9-correlated genes suggested the involvement of DUSP9 in cell adhesion, wound healing, cell migration, transcription regulation and metabolic process. Furthermore, DUSP9 expression in tumor tissues exhibited an inverse relationship with immune cell infiltration within the tumor microenvironment (TME). In conclusion, this study provided evidence that DUSP9 was up-regulated in HNSCC tissues and may play a pivotal role in HNSCC progression, suggesting its potential as a novel biomarker.

## Introduction

Head and neck cancers represent the sixth most common cancer globally. Head and neck squamous cell carcinoma (HNSCC) accounts for the majority of these cancers and originates from the squamous epithelium of the oral cavity, oropharynx, larynx, and hypopharynx. HNSCC is notorious for its poor prognosis and high heterogeneity 1, 2. The primary risk factors include tobacco and alcohol consumption, exposure to environmental carcinogens, and human papillomavirus (HPV) infection 3. Curative therapies encompass surgery, radiation, chemotherapy, and immunotherapy, tailored to the anatomical subsite, disease stage, and patient preferences 3. Despite optimal treatment, HNSCC patients face a grim prognosis, with a 50% overall five-year survival rate 4. To date, there is an absence of reliable, clinically relevant prognostic or predictive biomarkers in HNSCC.

The dual specificity phosphatase (DUSP) family represents the largest group of protein phosphatases that modulate the activity of mitogen-activated protein kinases (MAPKs). These phosphatases specifically dephosphorylate threonine and/or tyrosine residues within the kinase activation loop's T-X-Y motif 5. All DUSPs possess a conserved phosphatase domain containing essential Asp, Cys, and Arg residues that form the catalytic site 6. DUSPs containing the KIM domain are generally classified as typical DUSPs or MAP kinase phosphatases (MKPs), while those lacking this domain are considered as atypical DUSPs 6. As regulators of MAPK signaling, DUSPs modulate critical signaling pathways disrupted in various diseases 7. DUSP9, also known as MAP kinase phosphatase 4 (MKP4), was initially identified in 1997 8. It exhibits broad dephosphorylation specificity for MAPK substrates, including JNK, p38, and ERK1/2, with a marked preference for ERK kinases 8. Recent studies have implicated DUSP9 in the progression of several cancers, with its upregulation observed in hepatocellular and breast carcinomas, promoting tumor growth 9, 10. Conversely, DUSP9 downregulation has been noted in clear cell renal carcinoma, gastric carcinoma, and colorectal carcinoma, functioning as a tumor suppressor 11-13. However, the role and regulatory mechanisms of DUSP9 in HNSCC await further investigation.

In our study, DUSP9 exhibited increased expression in HNSCC tissues relative to normal samples, as evidenced by both TCGA data and clinical specimens. The expression of DUSP9 escalated with advancing tumor grade and stage, emerging as an independent prognostic factor associated with poor outcomes in HNSCC. DUSP9 deficiency inhibited the proliferation and migration of HNSCC cells, while DUSP9 overexpression increased their proliferative and migratory abilities, further supporting the phenotype of promoting tumor growth and metastasis of DUSP9 in HNSCC. DUSP9 expression was predominantly localized to malignant cells within HNSCC tissues and more concentrated in metastatic tumors than primary tumors. Functional enrichment analysis revealed that the DUSP9-correlated gene network was implicated in cell adhesion, transcription, wound healing and cell migration, which suggested a mechanism for tumor progression. Estimation of immune infiltration showed a negative correlation between DUSP9 expression and levels of immune cell infiltration, including T cells, B cells, monocytes, macrophages, neutrophils and dendritic cells.

## Materials and Methods

### Public database utilization

The Cancer Genome Atlas (TCGA, https://www.cancer.gov/tcga) 14 initiative provides a treasure trove of clinical data and RNA-seq results for primary cancers and matched normal samples, serving as a foundational resource for cancer research. The expression data and clinical information of HNSCC from TCGA were downloaded from University of California Santa Cruz (UCSC, https://xena.ucsc.edu/) 15 for grouped and visualized. The survival curves and correlation between genes utilizing TCGA data were depicted via Gene Expression Profiling Interactive Analysis (GEPIA2, http://gepia2.cancer-pku.cn/) 16. Proteomics data, derived from the Clinical Proteomic Tumor Analysis Consortium (CPTAC, https://pdc.cancer.gov/pdc/) 17 program, were presented via The University of Alabama at Birmingham Cancer data analysis Portal (UALCAN, https://ualcan.path.uab.edu/) 18. The Database for Annotation, Visualization and Integrated Discovery (DAVID, https://david.ncifcrf.gov/) 19 was used for the functional enrichment analysis of the gene sets of interest. TIMER (http://timer.cistrome.org) 20 facilitated the analysis of immune cell infiltration within the tumor microenvironment.

### Single cell RNA sequencing data and analysis

The scRNA-seq datasets for human HNSCC samples were sourced from GSE103322 21 and GSE234933 22. GSE103322 profiled 5,902 single cells from 18 patients with oral cavity tumors, including primary tumor samples from 18 patients and matching lymph node metastatic samples from 5 of these patients. GSE234933 profiled 187,399 single cells from 51 patients with multiple types of HNSCC primary sites including oral cavity, larynx/hypopharynx, oropharynx, and nasopharynx. The scRNA-seq data were analyzed using the R package Seurat (version 3.1.2) 23, following a standardized pipeline as previously described 24. Cell types were annotated according to the original papers, with results visualized using the DimPlot, DotPlot and VlnPlot functions within the Seurat package. Tumor cell clusters from GSE234933 were identified and used for a Pearson correlation analysis based on gene expression.

### Human HNSCC samples collection

Head and neck squamous cell carcinoma (HNSCC) tissue samples were collected from Beijing Tongren Hospital between January and December of 2023. Both tumor and paratumor tissues were included. Patients diagnosed with primary squamous cell carcinoma of the nasal cavity, paranasal sinuses, oropharynx, hypopharynx, or lower pharynx who had surgical specimens retained during the operation were considered for inclusion in the study. Patients who had previously undergone surgery for squamous cell carcinoma of the head and neck or other areas, or for inverted papilloma, were excluded. A total of 48 cases were collected and consisted of 43 men and 5 women ranging in age from 42 to 87 years old. Forty samples received no adjuvant therapy before surgery. Three samples received induction chemotherapy and immunotherapy before surgery, and one sample received induction chemotherapy before surgery. The detailed clinical parameters of these samples are shown in Table S1. This research was approved by the Ethics Committee of BeiJing TongRen Hospital (TREC2023-KY073). All participants involved in this study have signed informed consent.

### Immunohistochemistry (IHC)

Tissue samples from HNSCC patients were fixed in 4% paraformaldehyde, embedded in paraffin, and sectioned into 4 μm slices. Dehydration of paraffin sections was followed by treatment with 3% H_2_O_2_ for 30 minutes, then incubation with ImmunoBlock reagent for an additional 30 minutes at room temperature. Sections were incubated overnight at 4°C with primary antibodies against DUSP9 (FNab02571, FineTest). Subsequent treatment with secondary antibodies (GK600705, GeneTech) was carried out for 30 minutes at room temperature. Finally, sections were stained with hematoxylin for 5 minutes and developed using a developer for 1 minute. A DAB-based measuring standard was applied for quantitative analysis of DUSP9 mean density using ImageJ software (National Institutes of Health, NIH), with five randomly selected fields of view (20×) analyzed for quantification.

### Western blot

Tissue samples were minced, lysed, and homogenized in radio-immunoprecipitation assay (RIPA) buffer (Beyotime) at low temperature for 30 minutes. The lysate was centrifuged at 12,000 rpm for 10 minutes, and protein concentration was determined using the BCA Protein Assay Kit (Pierce). Proteins were resolved by SDS-PAGE and transferred onto nitrocellulose membranes (Amersham). After blocking, membranes were probed with primary antibodies against DUSP9 (FNab02571, FineTest) and β-actin (TA-09, ZSGB-Bio), followed by incubation with corresponding secondary antibodies. Signals were detected using the LAS500 (GE, NY) and quantified with ImageJ software (National Institutes of Health, NIH).

### Cell lines and cell culture

The human HNSCC cell line CAL27 (ATCC, CRL-2095) was preserved and passaged by the Key Laboratory of Otolaryngology Head and Neck Surgery of Beijing Tongren Hospital. CAL27 cells were cultured in Dulbecco's modified Eagle medium (DMEM, SH30022.01, Cytiva) containing 10% fetal bovine serum (FBS, HK-CH500, HUANKE) and 1% penicillin and streptomycin (P1400, Solarbio) in a humidified atmosphere of 5% CO_2_ at 37°C. Another human HNSCC cell line FaDu (CL-0083) was purchased from Procell (Wuhan, China). FaDu cells were cultured in minimum essential medium (MEM, SH30024.01, Cytiva) containing 10% FBS and 1% penicillin and streptomycin in a humidified atmosphere of 5% CO_2_ at 37°C.

### RNA interference sequences and plasmids

Small interfering RNAs (siRNAs) targeting DUSP9 were designed and synthesized by Tsingke (China). The sequences of si*DUSP9* were 1#: 5'-CGACUGCUCUGAUGCGGAATT-3' and 2#: 5'-CUUCAGCAGAUUCCAAGGCCGA-3', and the sequence of negative control siRNA (siNC) was 5'-UUCUCCGAACGUGUCACGUTT-3'. The siRNAs were transfected separately into cells using Lipofectamine RNAiMAX (Invitrogen) according to the manufacturer's instructions.

The mammalian expression vector for DUSP9 overexpression was constructed using the pcDNA3.1-Myc-His vector (Invitrogen). The plasmids were transfected separately into cells using jetPRIME (101000046, Polyplus) according to the manufacturer's instructions.

### Cell proliferation assay

Cells were transfected with siRNAs or plasmids described as above. After 24 hours, cells were detached and counted, then plated at a density of 2,000 cells per well in a 96-well plate. The relative cell viability was measured in optical density (OD) at 450 nm and 630 nm every day with the use of Cell Counting Kit-8 (CCK-8, K1018, APExBIO).

### Wound healing assay

Cells were plated at a density of 1×10^6^ cells per well in a 6-well plate (approximately 80-90% confluence) and transfected with siRNAs or plasmids described as above. After 24 hours, the wounds were created by scratch artificially and the cells were treated with serum-free medium. Images were captured with BZ-X800 instrument (Keyence). The ratio of the wound healing areas was measured using ImageJ software (National Institutes of Health, NIH).

### Statistical analysis

All experimental data were analyzed and graphed using GraphPad Prism 8.0 (Inc. San Diego, CA, USA). Data were presented as means ± SEM for the biological replicates. A two-tailed paired Student's t-test was conducted to assess significant differences between two paired groups. A two-tailed unpaired Student's t-test was used for two unpaired groups. A one-way ANOVA test was used for multiple groups. A *p*-value of less than 0.05 was considered statistically significant. Univariate analyses of the clinical features contributing to DUSP9 expression were performed using a Student's t-test for binary categorical variables and a one-way ANOVA for multiple categorical variables by SPSS 22.0 (Inc. IBM, NY, USA).

## Results

### Public data indicate upregulation and prognostic implications of DUSP9 in HNSCC

**Figure 1:**
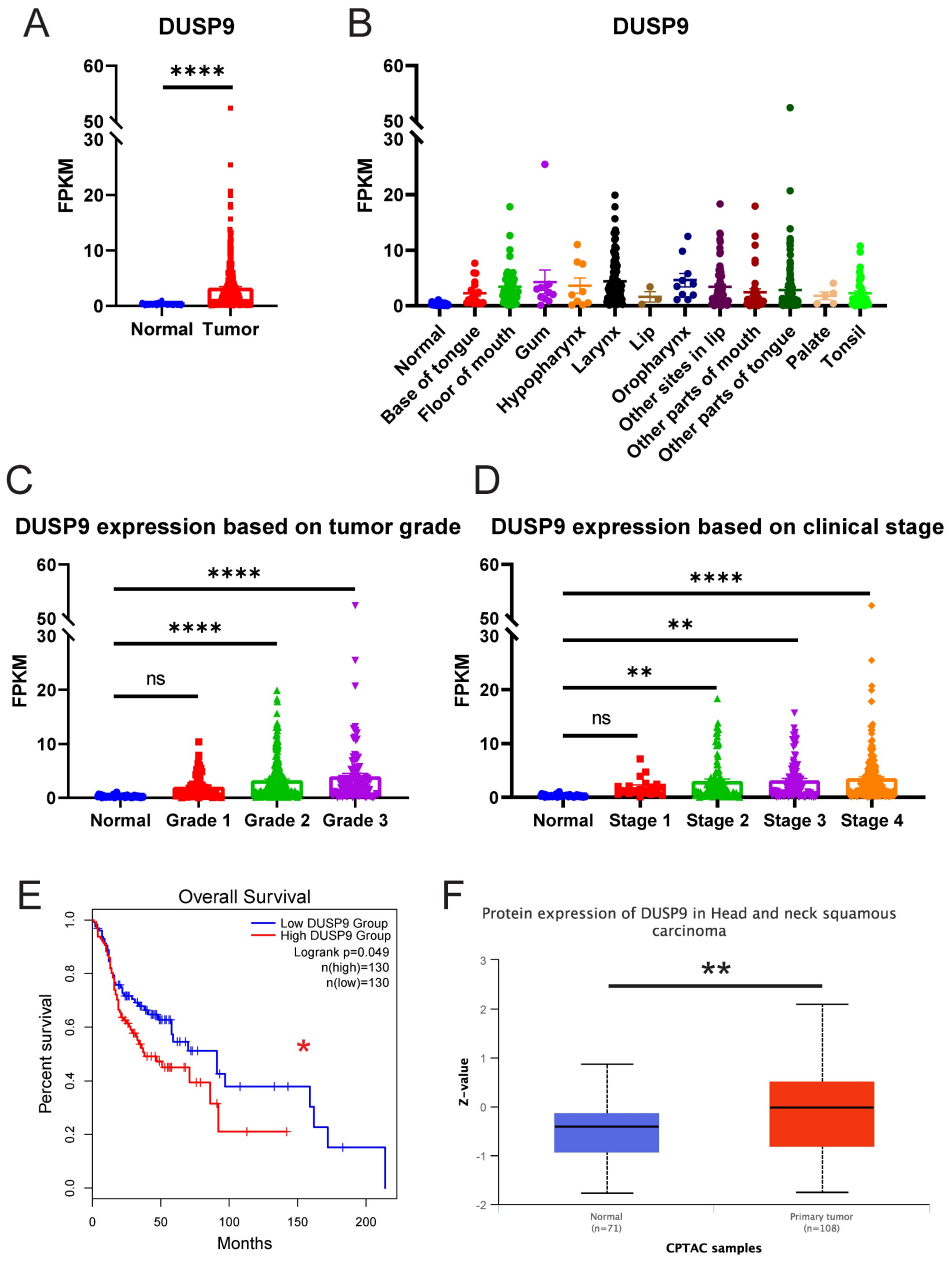
** DUSP9 is up-regulated in HNSCC as reflected in public data.** (A) The mRNA expression profiles of DUSP9 in HNSCC based on sample types from TCGA. (B) The mRNA expression profiles of DUSP9 in HNSCC based on primary tumor tissues from TCGA. (C) The mRNA expression profiles of DUSP9 in HNSCC based on tumor grade from TCGA. Grade 1, well differentiated (low grade); Grade 2, moderately differentiated (intermediate grade); Grade 3, poorly differentiated (high grade). (D) The mRNA expression profiles of DUSP9 in HNSCC based on clinical stages from TCGA. (E) Kaplan-Meier curve of HNSCC patients' survival grouped by DUSP9 mRNA expression levels. (F) The protein expression levels of DUSP9 in HNSCC based on sample types from CPTAC. Data were shown as mean ± SEM (A-D); p-value was determined by Student's t-test (A), one-way ANOVA test (C, D); others according to the original websites (E, F); ns, not significant; * p < 0.05; ** p < 0.01; **** p < 0.0001.

To explore the clinical significance of DUSP9 in HNSCC, we analyzed the relative expression of DUSP9 in HNSCC patients from TCGA data source, which currently comprises 522 tumor samples and 44 normal or paratumor samples. The results showed the mRNA expression level of DUSP9 was significantly elevated in tumor tissues (Figure 1A). Given the complexity of HNSCC tumor subtypes, we grouped the samples based on the tumor tissue source and visualized for DUSP9 expression (Figure 1B). Compared with the normal head and neck tissue, DUSP9 was highly expressed in all types of HNSCC, and there is no obvious difference among all types. Further analysis indicated that DUSP9 expression increased with tumor grade progression (Figure 1C). Additionally, DUSP9 expression escalated with individual cancer stage in the early phases (Figure 1D). Survival analysis suggested that patients with elevated DUSP9 expression faced a poorer prognosis compared to those with lower expression levels (Figure 1E). The CPTAC dataset, which includes 10 cancer proteomics cohorts of prospectively collected tumors, currently encompasses 71 normal samples and 108 primary tumor samples for HNSCC 25. Consistent with mRNA findings, DUSP9 protein expression was also up-regulated in tumor samples (Figure 1F). Taken together, these results suggested that the expression of DUSP9 is increased in human HNSCC tissues and correlated with the progression and poor prognosis of HNSCC patients.

### Clinical data confirm upregulated DUSP9 expression in HNSCC tumor tissues

**Figure 2:**
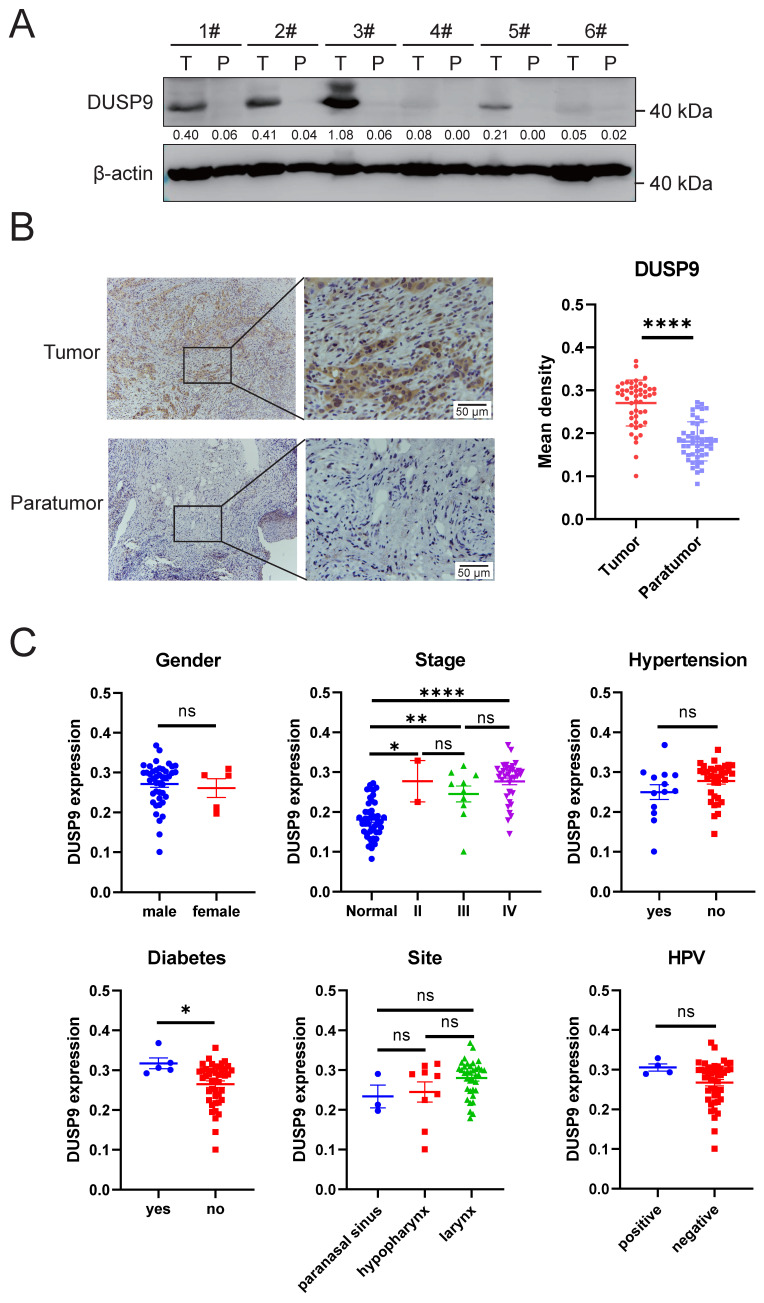
** Clinical data demonstrated that DUSP9 was highly expressed in HNSCC tumor tissues.** (A) Detection of DUSP9 expression in clinical tumor (T) and paratumor (P) samples by Western blot. The values between the two bands represent the DUSP9/β-actin ratio as measured by ImageJ. (B) Detection of DUSP9 expression in clinical tumor and paratumor samples by IHC. Mean density was quantified using ImageJ (n=48). Data were shown as mean ± SEM; p-value was determined by paired Student's t-test; **** < 0.0001. (C) DUSP9 expression in samples grouped by clinical parameters, including gender, stage, hypertension, diabetes, site and HPV infection. Data were shown as mean ± SEM; p-value was determined by Student's t-test for two groups or one-way ANOVA test for multiple groups; ns, not significant; * p < 0.05; ** p < 0.01; **** p < 0.0001.

We further examined DUSP9 protein expression in clinical samples, including primary tumor and matched paratumor tissues. Western blot result showed that DUSP9 was higher in HNSCC tissues than paratumor tissues (Figure 2A). Immunohistochemical analysis of DUSP9 staining in tumor tissues from 48 HNSCC patients confirmed significantly increased DUSP9 expression in tumor tissues compared to paratumor tissues (Figure 2B). In aggregate, these findings underscore the significant upregulation of DUSP9 in HNSCC, implicating it in tumorigenesis and poor prognosis, and warranting further exploration as a potential disease marker.

We also analyzed the various clinical features contributing to DUSP9 expression in tumor tissues. As shown in Table 1 and Figure 2C, the expression of DUSP9 was significantly higher in HNSCC patients with a history of diabetes than in those without. According to the bioinformatics analysis (Figure 1D), DUSP9 expression and difference significance increased with the progression of the clinical stage (Figure 2C). In addition to the univariate analysis presented in Table 1, multiple linear regression was employed to examine the relationship between clinical features and DUSP9 expression. It was also found that diabetes was the only independent variable significantly associated with increased DUSP9 expression in tumors (*p* = 0.009).

### DUSP9 knockdown reduces cell proliferation and migration in HNSCC cells

**Figure 3:**
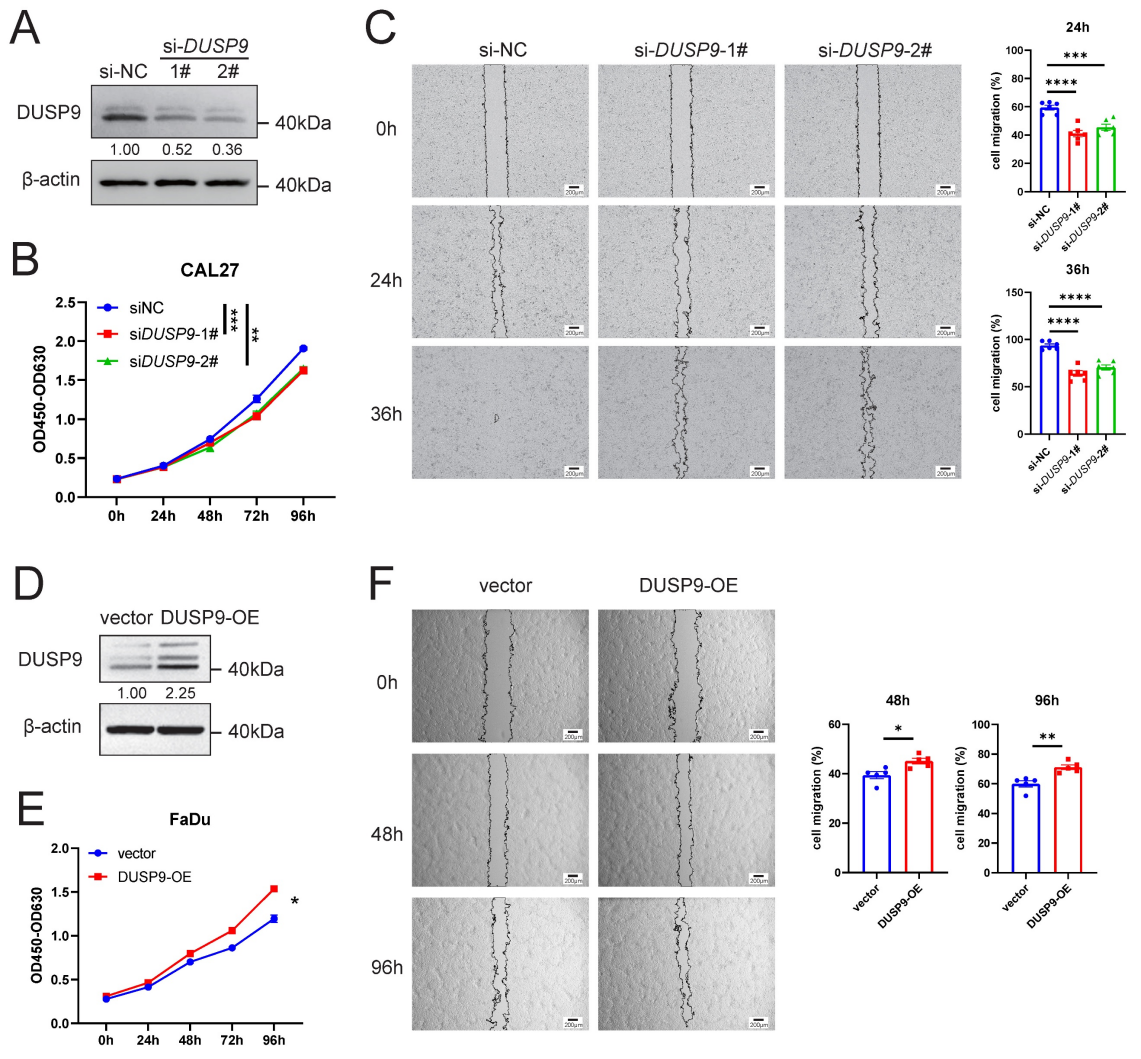
** DUSP9 increased HNSCC cell proliferation and migration.** (A) Detection of DUSP9 knockdown efficiency in CAL27 cells using siRNA by Western blot. The values between the two bands represent the DUSP9/β-actin ratio as measured by ImageJ. (B) Cell proliferation was determined using CCK-8 assay for CAL27 cells with DUSP9 knockdown. (C) Wound healing assay to examine the migration ability of CAL27 cells with DUSP9 knockdown. The healed areas were measured using ImageJ and calculated for percentage of cell migration. (D) Detection of DUSP9 overexpression efficiency in FaDu cells by Western blot. The values between the two bands represent the DUSP9/β-actin ratio as measured by ImageJ. (E) Cell proliferation was determined using CCK-8 assay for FaDu cells with DUSP9 overexpressed. (F) Wound healing assay to examine the migration ability of FaDu cells with DUSP9 overexpressed. The healed areas were measured using ImageJ and calculated for percentage of cell migration. Data were shown as mean ± SEM; p-value was determined by one-way ANOVA test (B, C) or Student's t-test (E, F); * p < 0.05, ** p < 0.01, *** p < 0.001, **** p < 0.0001.

To further investigate the function of DUSP9 in HNSCC progression, we used siRNAs to knockdown DUSP9 in CAL27 cells. The efficiency of knockdown was validated by Western blot analysis (Figure 3A). CCK8 assay indicated that DUSP9 knockdown inhibited HNSCC cell proliferation (Figure 3B). Moreover, we performed wound healing assays to investigate whether DUSP9 affects HNSCC cell migration, and found that DUSP9 knockdown significantly delayed wound healing (Figure 3C).

We also overexpressed DUSP9 by plasmids in FaDu cells, and the overexpression efficiency was shown in Figure 3D. DUSP9 overexpression (OE) increased HNSCC cell proliferation by CCK8 assay (Figure 3E) and improved cell migration by wound healing assay (Figure 3F). Collectively, these results suggested that DUSP9 had an integrated promotional role in regulating HNSCC progression.

### DUSP9 is specifically expressed in malignant cells and correlated with cell migration

**Figure 4:**
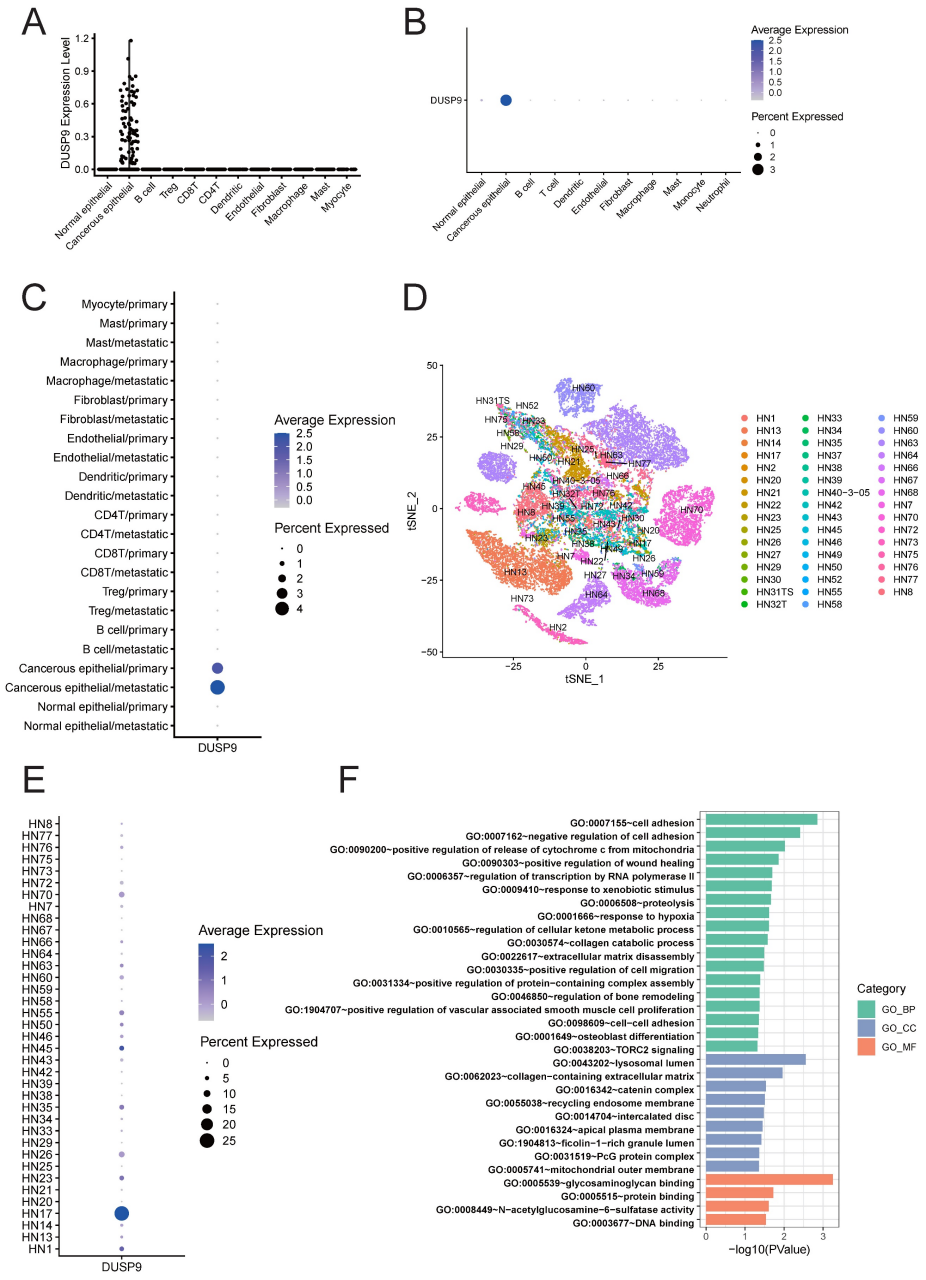
**Exclusive expression of DUSP9 in malignant cells and its correlation network involved in the regulation of cell migration.** (A) Scatter plot showed the expression of DUSP9 across different cell types as determined by scRNA-seq data from GSE103322. (B) Dot plot showed the expression of DUSP9 across different cell types as determined by scRNA-seq data from GSE234933. (C) Dot plot showed the expression of DUSP9 based on different cell types and tissue sources as determined by scRNA-seq data from GSE103322. (D) Cancerous epithelials grouped by sample idents for t-SNE reduction from GSE234933. (E) Dot plot showed the expression of DUSP9 across different sample idents in cancerous epithelials. The groups whose cell counts less than 50 were not shown. (F) GO enrichment analysis of DUSP9-correlated genes based on biological process (BP), cellular component (CC) and molecular function (MF). The x-axis represents -log10(p-value) while the y-axis represents the term of GO.

Given the complexity of tumor tissue composition, which includes tumor epithelial cells, fibroblasts, endothelial cells, and various immune cells, we analyzed cell type-specific DUSP9 expression using HNSCC scRNA-seq data from GSE103322 21 and GSE234933 22. After quality control, a total of 5,846 single cells in GSE103322 and 186,430 single cells in GSE234933 were separately categorized into clusters representing the predominant cell types within tumor tissues. Both datasets indicated that DUSP9 expression was confined to tumor cells and was virtually undetectable in immune cells and stromal cells (Figure 4A&B). Considering the limited number of genes detected in single-cell RNA-seq, we examined the bulk RNA-seq data from the DICE database (https://dice-database.org/genes/DUSP9) 26 and our previous analysis of T cells, B cells, monocytes and NK cells 24, reaffirming that DUSP9 is indeed hardly expressed in immune cells.

During tumor progression, primary malignant tumors may initiate invasion-metastasis cascade to disseminate to distant organs forming metastatic lesions, which is the main cause of failure in tumor treatment. Using the scRNA-seq data from GSE103322, we added the classification based on tissue sources from primary tumors (oral cavity) versus metastatic tissues (lymph node) to previous cell type classification. The dot plot also showed the absolute predominant expression of DUSP9 in tumor epithelial cells, in addition, the new finding was that DUSP9 was higher expressed in metastatic tissues than primary tumors (Figure 4C). This result suggested that DUSP9 may be involved in the metastatic development of HNSCC.

For functional analysis, cancer epithelial cell subsets were extracted from scRNA-seq dataset GSE234933. We showed the clusters based on sample idents (Figure 4D), and found that DUSP9 was high expressed in HN17 sample (Figure 4E). Consequently, cancer epithelial cells from HN17 sample were extracted for gene expression correlation analysis. A total of 428 genes exhibiting significant correlation (r > 0.20) with DUSP9 were identified using Pearson correlation analysis (Table S2). The genes were then subjected to Gene Ontology (GO) enrichment analysis. The results indicated that the gene set was predominantly enriched in biological processes that related to cell adhesion, wound healing, transcription by RNA polymerase II, collagen catabolic process, extracellular matrix disassembly, cell migration and so on (Figure 4F, Table S3), suggesting that DUSP9 may contribute to tumor progression by promoting tumor cell proliferation and the ability to migrate to other tissues.

### DUSP9 was negatively related to immune cells infiltration

**Figure 5:**
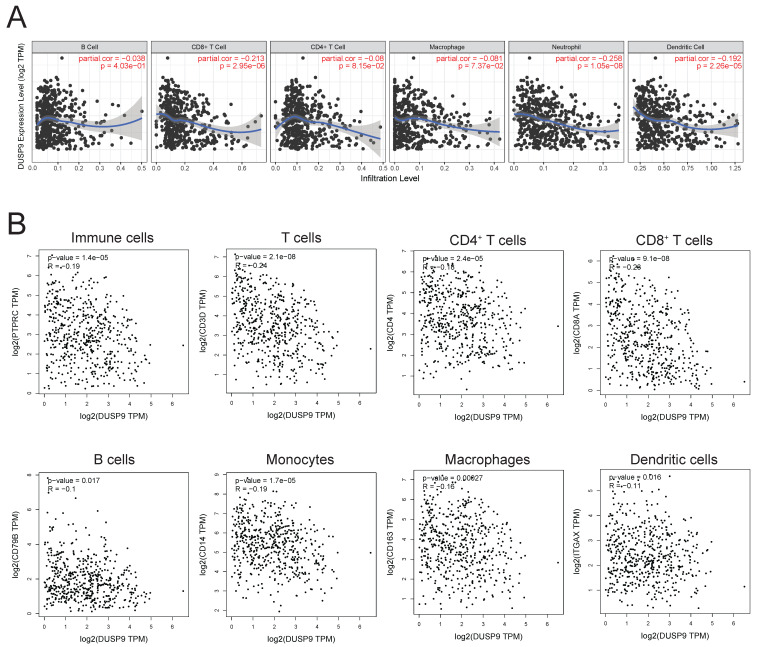
** DUSP9 negative correlation with immune infiltration.** (A) Correlation between DUSP9 expression levels and immune cell infiltration levels in human HNSCC as calculated by TIMER. (B) Correlation between DUSP9 expression levels and immune cell markers expression levels in human HNSCC from GEPIA2.

The tumor immune microenvironment exerts a profound influence on tumor growth, progression, and metastasis. Investigating the impact of DUSP9 on immune cells in tumor microenvironment is crucial for elucidating the mechanisms underlying head and neck cancer. Immune infiltration analysis in HNSCC using the TIMER database highlighted a negative association between DUSP9 expression in tumor tissues and the extent of immune cell infiltration, particularly CD8^+^ T cells, neutrophils, and dendritic cells (Figure 5A). Additionally, we also analyzed the correlation of DUSP9 using TCGA-derived HNSCC cohort data with immune cell marker molecules, including CD45 (immune cells), CD3/CD4/CD8 (T cells), CD79B (B cells), CD14 (monocytes), CD163 (macrophages) and CD11c (dendritic cells). The expression level of DUSP9 was negatively correlated with the expression level of the aforementioned immune cell marker molecules (Figure 5B). These findings suggested that DUSP9 may also modulate tumor development by shaping the tumor microenvironment.

## Discussion

DUSP9 can dephosphorylate its substrates to negatively regulate their activity, including the MAPK (ERK/JNK/p38), mTOR and ASK1 11, 12, 27, 28. Previous studies showed that DUSP9 was directly involved in the development of multiple tumors, including hepatocellular carcinoma, breast cancer, renal carcinoma, gastric carcinoma and colorectal carcinoma 29. Our study demonstrates that DUSP9 is up-regulated to promote HNSCC and associated with poor prognosis as a potential disease marker for the first time.

The high mortality rate among HNSCC patients, reaching up to 50%, underscores the urgency to develop more effective therapeutic strategies. By comparing the expression of DUSP9 in HNSCC tumors and paratumor tissues, we found DUSP9 to be significantly up-regulated in tumors. In contrast, DUSP9 expression was negligible in normal head and neck tissues. The scRNA-seq analysis further demonstrated that DUSP9 expression was confined to tumor epithelial cells, with minimal expression in fibroblasts, endothelial cells or immune cells. This selective expression pattern in tumor cells suggests that DUSP9 could be a promising therapeutic target in HNSCC. Interestingly, in the detection of clinical samples (Figure 2A) and in the analysis of scRNA-seq sample clusters (Figure 4C), DUSP9 showed relatively different expression levels in different patients, which suggests that DUSP9 is a highly plastic gene in HNSCC tumors and may be used for molecular classification of HNSCC tumors 24, 30. Combined with its expression correlated with tumor grade and clinical stage (Figure 1C&D), DUSP9 was expected to server as a clinical marker molecule in HNSCC to indicate the disease process and assist clinical treatment. In addition, in the clinical factors analysis, we found that DUSP9 expression was higher in patients with a history of diabetes than in those without (Figure 2C, Table 1), which may be related to the role of DUSP9 in insulin resistance 31.

Both tumor cell function experiments and related gene enrichment analysis were suggested that DUSP9 contributed to tumor progression, promoting tumor cell proliferation and migration. The positively correlated genes, such as MYLIP, SNCG, NCOR2, BGN and ALDH3A1, have been found involved in progression of multiple types of tumors 32-36. Moreover, the expression levels of some positively correlated genes, such as ACIN1, NCOR2, BGN, RIN2, HSPG2 and LRIG3, were also increased in HNSCC according to the public data (https://ualcan.path.uab.edu). These results further supported that DUSP9 increased the ability of HNSCC tumor cells in proliferation, local invasion and migration, suggesting a promoting role in HNSCC tumor progression.

DUSP9 also plays a role in regulating the tumor immune microenvironment. Tumor cells can shape the microenvironment by secreting cytokines and expressing immunosuppressive molecules. For example, tumor-derived cytokines G-CSF and IL-1 can lead to the formation of an immunosuppressive phenotype and induce immune escape 37, 38. Tumor-derived cytokines, chemokines, and even metabolic conditions (e.g., pH, oxygen levels, and nutrient availability) can all modulate immune cell function within the TME. Therefore, it is plausible that DUSP9 promotes the development of head and neck tumors through dual effects on malignant cells and tumor microenvironment.

Our study still has some limitations. Due to the small size of clinical samples in our research, we couldn't confirm the relationship between DUSP9 expression level and patient survival in clinical. Further research is warranted to elucidate the specific mechanisms by which DUSP9 functions within HNSCC tumors and their microenvironment.

In conclusion, our study demonstrates that DUSP9 is up-regulated in human HNSCC and promotes tumor progression, suggested that DUSP9 may serve as a biomarker for HNSCC prognosis and a potential target for HNSCC treatment.

## Supplementary Material

Supplementary table 1.

Supplementary table 2.

Supplementary table 3.

## Figures and Tables

**Table 1:** Univariate analyses of clinical features contributing to DUSP9 expression

Factor		N=48	Percent	*P* value
Gender	male	43	89.6%	0.687
	female	5	10.4%	
Age				0.885
Stage	II	2	4.2%	0.149
	III	10	20.8%	
	IV	36	75.0%	
Grade	low	10	20.8%	0.166
	low-medium	7	14.6%	
	medium	28	58.3%	
	high	3	6.3%	
Site	paranasal sinus	3	6.3%	0.099
	larynx	36	75.0%	
	pharynx	9	18.8%	
Surgery	no	0	0%	#
	yes	48	100.0%	
Immunotherapy	no	45	93.8%	0.06
	yes	3	6.3%	
Chemotherapy	no	44	91.7%	0.135
	yes	4	8.3%	
Pulmonary Embolism	no	43	89.6%	0.88
	yes	5	10.4%	
Hypertension	no	35	72.9%	0.109
	yes	13	27.1%	
Diabetes	no	43	89.6%	**0.037**
	yes	5	10.4%	
Coronary Disease	no	45	93.8%	0.696
	yes	3	6.3%	
Smoking	no	17	35.4%	0.185
	yes	31	64.6%	
Alcohol	no	20	41.7%	0.215
	yes	28	58.3%	
HPV	HPV (-)	44	91.7%	0.171
	HPV (+)	4	8.3%	

# Unable to compute due to numerical problems

## Abbreviations

HNSCC: Head and neck squamous cell carcinoma
DUSP9: Dual specificity phosphatase 9
scRNA-seq: Single-cell RNA sequencing
TME: tumor microenvironment
HPV: human papillomavirus
DUSP: Dual specificity phosphatase
MAPKs: mitogen-activated protein kinases
MKPs: MAP kinase phosphatases
MKP4: MAP kinase phosphatase 4
TCGA: The Cancer Genome Atlas
UCSC: University of California Santa Cruz
GEPIA2: Gene Expression Profiling Interactive Analysis
CPTAC: Clinical Proteomic Tumor Analysis Consortium
UALCAN: University of Alabama at Birmingham Cancer data analysis Portal
DAVID: Database for Annotation, Visualization and Integrated Discovery
IHC: immunohistochemistry
NIH: National Institutes of Health
ATCC: American Type Culture Collection
DMEM: Dulbecco's modified Eagle medium
MEM: minimum essential medium
FBS: fetal bovine serum
siRNAs: small interfering RNAs
siNC: negative control siRNA
OD: optical density
CCK-8: Cell Counting Kit-8
OE: overexpression
GO: Gene Ontology
MYLIP: myosin regulatory light chain interacting protein
SNCG: synuclein gamma
NCOR2: nuclear receptor corepressor 2
BGN: biglycan
ALDH3A1: aldehyde dehydrogenase 3 family member A1
ACIN1: apoptotic chromatin condensation inducer 1
RIN2: Ras and Rab interactor 2
HSPG2: heparan sulfate proteoglycan 2
LRIG3: leucine rich repeats and immunoglobulin like domains 3
t-SNE: t-distributed stochastic neighbor embedding
